# ﻿A new species of *Sinopoda* from China, with first description of the male of *S.wuyiensis* Liu, 2021 (Araneae, Sparassidae)

**DOI:** 10.3897/zookeys.1116.85303

**Published:** 2022-08-09

**Authors:** Yang Zhong, Meng-Yun Zeng, Chao-Lan Gu, Hui-Liang Yu, Jing-Yuan Yang

**Affiliations:** 1 School of Nuclear Technology and Chemistry & Biology, Hubei University of Science and Technology, Xianning 437100, Hubei, China; 2 Hubei Key Laboratory of Radiation Chemistry and Functional Materials, Hubei University of Science and Technology, Xianning 437100, Hubei, China; 3 Shennongjia National Park Administration, Shennongjia 442421, Hubei, China; 4 Hubei Province Key Laboratory of Conservation Biology for Shennongjia Golden Monkey, Shennongjia 442421, Hubei, China

**Keywords:** Biodiversity, Fujian, Hubei, huntsman spiders, taxonomy

## Abstract

One new species of the genus *Sinopoda* Jäger, 1999, *S.muyuensis***sp. nov.** (♂, ♀), is described and figured from the Shennongjia Forestry District, Hubei Province, China. In addition, the male of *Sinopodawuyiensis* Liu, 2021 is described for the first time from the Wuyishan National Nature Reserve, Fujian Province, China.

## ﻿Introduction

The genus *Sinopoda* was established by Jäger (1999). As currently recognised, it comprises 137 species, of which 71 species were recorded from China, representing 51.8% of the global species [World Spider Catalog (WSC) 2022]. The genus has been reported from East Asia (85 species in China, Japan, and Korea), Southeast Asia (50 species in Brunei, Indonesia, Laos, Malaysia, Myanmar, Thailand, and Vietnam) and South Asia (a single species in India) (Zhang et al. 2021).

We have found two *Sinopoda* species present in collections from China in the past year. The discovery of one of these species, *Sinopodamuyuensis* sp. nov. from Hubei represents the first record of this species in Shennongjia Forestry District. Furthermore, specimens of *Sinopodawuyiensis* Liu, 2021 from Fujian have allowed us to provide the first description of the male of the species.

## ﻿Materials and methods

Specimens were examined and measured with an Olympus SZX7 stereomicroscope. Positions of tegular appendages are given according to clock positions, based on the left palp in ventral view. Male and female copulatory organs were examined and illustrated after dissection from the spider bodies; vulvae were cleared in a warm 10% potassium hydroxide (KOH) solution. All photographs were captured with a KUY NICE industrial digital camera (20.0 megapixels) mounted on an Olympus CX43 dissecting microscope, and assembled using Helicon Focus 3.10.3 image stacking software. Photographic images were then edited using Adobe Photoshop CC 2018. All measurements were obtained using an Olympus SZX7 stereomicroscope and are given in millimetres (mm).

Leg measurements are shown as: total length (femur, patella, tibia, metatarsus, tarsus). Number of macrosetae is listed for each segment in the following order: prolateral, dorsal, retrolateral, ventral (in femora and patellae ventral spines are absent and fourth digit is omitted in the setation formula). Abbreviations used in the text and figures are given below: **AB** = anterior bands, **ALE** = anterior lateral eye, **AME** = anterior median eye, **AW** = anterior width of carapace, **C** = conductor, **CH** = clypeus heightclypeus height, **dRTA** = dorsal branch of RTA, **E** = embolus, **EA** = embolic apophysis, **FD** = fertilization duct, **FE** = femur, **GA** = glandular appendage, **LL** = lateral lobes, **LS** = lobal septum, **MS** = membranous sac, **Mt** = metatarsus, **OL** = opisthosoma length, **OW** = opisthosoma width, **Pa** = patella, **PI** = posterior incision of LL, **PL** = carapace length, **PLE** = posterior lateral eyes, **PME** = posterior median eyes, **Pp** = palp or palpus, **PP** = posterior part of spermathecae, **PW** = carapace width, **RTA** = retrolateral tibial apophysis, **SP** = spermophor, **SS** = slit sensillum, **ST** = subtegulum, **T**= tegulum, **Ta** = tarsustarsus, **Ti** = tibia. I, II, III, IV–legs I to IV, **TP** = tegular protrusion, **vRTA** = ventral branch of RTA, **HUST** = School of Nuclear Technology and Chemistry and Biology, Hubei University of Science and Technology, Xianning, Hubei, China.

## ﻿Taxonomy

**Family Sparassidae Bertkau, 1872**

**Subfamily Heteropodinae Thorell, 1873**

### ﻿Genus Sinopoda

Taxon classificationAnimaliaAraneaeSparassidae

Jäger, 1999

E6C13599-7F3B-5283-9924-DFD323FBCC80

#### Type species.

*Sarotesforcipatus* Karsch, 1881

#### Diagnosis.

See Jäger (1999), Liu et al. (2008), Zhang et al. (2015), and Grall and Jäger (2020).

#### Composition.

Within the genus *Sinopoda*, five groups were established by Jäger and Ono (2000), Grall and Jäger (2020) and Zhang et al. (2021): *S.anguina*-group (*S.anguina* Liu, Li & Jäger, 2008, *S.bifurca* Grall & Jäger, 2020, *S.bispina* Grall & Jäger, 2020, *S.fornicata* Liu, Li & Jäger, 2008, *S.improcera* Zhong et al., 2019, *S.lata* Zhong et al., 2019, *S.longicymbialis* Grall & Jäger, 2020, *S.mamillata* Zhong, Cao & Liu, 2017, *S.nanphagu* Grall & Jäger, 2020, *S.phiset* Grall & Jäger, 2020, *S.rotunda* Grall & Jäger, 2020 and *S.tuber* Grall & Jäger, 2020); *S.chiangmaiensis*-group (*S.chiangmaiensis* Grall & Jäger, 2020, *S.lot* Grall & Jäger, 2020 and *S.phathai* Grall & Jäger, 2020); *S.globosa*-group (*S.globosa* Zhang, Zhang & Zhang, 2015, *S.longiducta* Zhang, Zhang & Zhang, 2015, *S.mi* Chen & Zhu, 2009, *S.ovata* Zhong et al., 2019, *S.triangula* Liu, Li & Jäger, 2008 and *S.yaanensis* Zhong et al., 2019); *S.tumefacta*-group (*S.crassa* Liu, Li & Jäger, 2008, *S.dehiscens* Zhong et al., 2019, *S.erromena* Zhong et al., 2019, *S.tumefacta* Zhong et al., 2019, *S.yanlingensis* Zhong et al., 2019 and *S.yaojingensis* Liu, Li & Jäger, 2008); *S.okinawana*-group [*S.forcipata* (Karsch, 1881), *S.cochlearia* Zhang, Zhang & Zhang, 2015, *S.derivata* Jäger & Ono, 2002, *S.fasciculata* Jäger Gao & Fei, 2002, *S.guangyuanensis* Zhong et al., 2018, *S.hamata* (Fox, 1937), *S.koreana* (Paik, 1968), *S.okinawana* Jäger & Ono, 2000, *S.tanikawai* Jäger & Ono, 2000, *S.wangi* Song & Zhu, 1999]. Ninety-six other species have not yet been grouped.

### ﻿ Sinopoda muyuensis sp. nov.

Taxon classificationAnimaliaAraneaeSparassidae

E2608A91-7CB4-5B24-BD5E-4EEBA1ECD61C

https://zoobank.org/4BCEBD12-8C2A-44EA-9180-DDE1FB4ACD39

**Figure 1.**
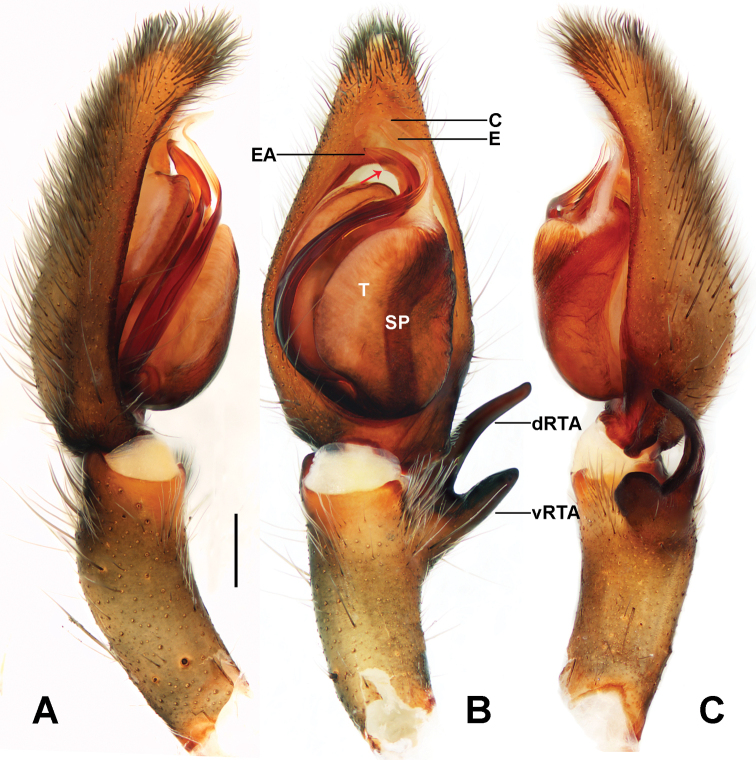
*Sinopodamuyuensis* sp. nov., holotype male **A–C** palp, left (**A** prolateral view **B** ventral view **C** retrolateral view). Abbreviations: C – conductor, dRTA – dorsal branch of RTA, E – embolus, EA – embolic apophysis, SP – spermophore, T – tegulum, vRTA – ventral branch of RTA. Red arrow: embolic apophysis projection. Scale bar: 0.5 mm.

**Figure 2.**
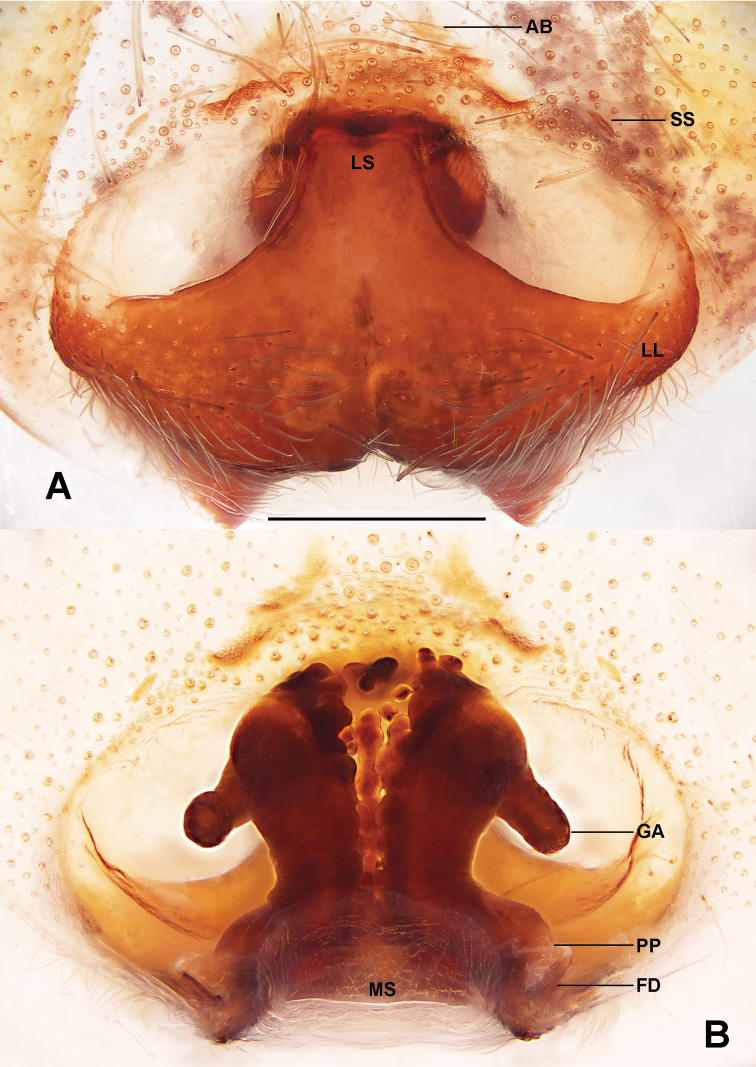
*Sinopodamuyuensis* sp. nov., paratype female **A** epigyne **B** vulva (**A** ventral view **B** dorsal view). Abbreviations: AB – anterior bands, FD – fertilisation duct, GA – glandular appendage, LL – lateral lobes, LS – lobal septum, MS – membranous sac, PP – posterior part of spermathecae, SS – slit sensillum. Scale bar: 0.5 mm.

Figs 1 , 2 , 4A, B, E–H , 5A, B , 6

#### Type material.

***Holotype***: ♂ (HUST 0003), **China: *Hubei***: Shennongjia Forestry District, Muyu Town, Guanmenshan Scenic Area; 31.45°N, 110.40°E; alt. 1200 m; 10.XII.2021; Y. Zhong leg. ***Paratypes*** (HUST): 2♂, 3♀, same data as holotype.

#### Etymology.

‘Muyu’ refers to the type locality of this species, Muyu Town.

#### Diagnosis.

The male of *Sinopodamuyuensis* sp. nov. resembles *S.angulata* Jäger, Gao & Fei, 2002 (Zhu et al. 2020: figs 1A–C, 2A–C) and *S.yichangensis* Zhu, Zhong & Yang, 2020 (Zhu et al. 2020: figs 4A–C, 5A–C; Gong and Zhong 2021: figs 2A–C) in having the embolus distally filiform, as long as the embolic apophysis, and RTA arising subdistally from tibia, but the new species can be separated from *S.angulata* by the posterior margins of the embolic apophysis being distinctly humped (smooth in *S.angulata*); from *S.yichangensis* by the tip of embolic apophysis with a pointed end (blunt in *S.yichangensis*). Females are similar to those of *S.angulata* (Zhu et al. 2020: fig. 2D, E) in having the epigyne with a lobal septum ~ 1/4 of the epigynal width, posterior part of the spermathecae considerably larger than the glandular projection, and the angle of the diverging internal duct system ~ 80°, but distinguished by the vulva with its internal duct system not touching (touching along the median line in *S.angulata*); glandular appendages extending into the median half of the internal duct system (anterior half in *S.angulata*); ends of internal duct system nearly straight in dorsal view (bent at 180° in *S.angulata*) (Figs 1, 2).

#### Description.

**Male.**PL 5.3, PW 4.7, AW 2.5, OL 5.8, OW 3.9. Eyes: AME 0.19, ALE 0.31, PME 0.22, PLE 0.32, AME–AME 0.20, AME–ALE 0.16, PME–PME 0.28, PME–PLE 0.36, AME–PME 0.38, ALE–PLE 0.25, CHAME 0.19, CHALE 0.26. Setation: Palp: 131, 101, 1100; Fe: I–III 323, IV 321; Pa: I–IV 101; Ti: I–IV 2326; Mt: I–II 1014, III 2026, IV 3036. Measurements of palp and legs: Palp 6.8 (2.4, 1.3, 1.3, –, 1.8), I 17.6 (4.4, 1.4, 5.5, 4.9, 1.4), II 19.1 (5.6, 1.7, 6.5, 3.9, 1.4), III 15.7 (4.9, 1.5, 4.8, 3.3, 1.2), IV 15.9 (5.3, 1.5, 4.8, 3.3, 1.0). Leg formula: II-I-IV-III. Cheliceral furrow with three anterior and four posterior teeth, and with ~ 36 denticles (Fig. 4A). Carapace yellowish brown dorsally, with yellow transversal stripe posteriorly, with shallow fovea and radial furrows. Chelicerae deep reddish brown. Sternum yellow with brown margin. Endites and labium deep yellowish brown, with margin deep brown. Legs yellowish brown, covered by short spines. Opisthosoma yellowish brown dorsally, with three pairs of dark patches laterally. Opisthosoma uniformly yellowish brown with some brown patches ventrally (Fig. 4E, F).

Palp as in Fig. 1. Cymbium distinctly longer than tibia. Embolus S-shaped, arising from tegulum at nearly the 6-o’ clock-position in ventral view. Conductor arising at 12- to 1-o’ clock-position from tegulum. RTA arising mesially to distally from tibia, with distinct brush of stiff setae. dRTA slender, finger-shaped, vRTA roughly rectangular in retrolateral view.

**Female.**PL 5.7, PW 4.8, AW 3.1, OL 5.9, OW 3.9. Eyes: AME 0.16, ALE 0.25, PME 0.18, PLE 0.25, AME–AME 0.27, AME–ALE 0.09, PME–PME 0.33, PME–PLE 0.35, AME–PME 0.32, ALE–PLE 0.28, CHAME 0.20, CHALE 0.25. Setation: Palp: 131, 101, 2026, 1014; Fe: I–III 323, IV 321; Pa: I–IV 000; Ti: I–III 2026, IV 2024; Mt: I–II 1014, III 2026, IV 3036. Measurements of palp and legs: Palp 5.8 (1.9, 0.8, 1.3, –, 1.8), I 14.6 (4.5, 2.4, 4.1, 2.3, 1.3), II 16.9 (5.2, 2.4, 4.1, 3.8, 1.4), III 13.2 (4.4, 2.0, 3.0, 2.6, 1.2), IV 14.2 (4.7, 1.8, 3.4, 3.0, 1.3). Leg formula: II-I-IV-III. Cheliceral furrow with three anterior and four posterior teeth, and with ~ 45 denticles (Fig. 4B).

Copulatory organ as in Fig. 2. Epigynal field wider than long, with short anterior bands and one slit sensillum on each side close to the epigynal field. Lateral lobes fused, with some fusion bubbles along median line. Fertilisation ducts arising posterolaterally. Membranous sac between fertilisation ducts almost rectangular.

Colouration in ethanol as in males, but generally slightly darker, Opisthosoma brown dorsally (Fig. 4G, H).

#### Distribution.

Known only from the type locality (Fig. 6).

### ﻿ Sinopoda wuyiensis

Taxon classificationAnimaliaAraneaeSparassidae

Liu, 2021

B2E27902-1E71-51EB-9C38-301039B35D63

**Figure 3.**
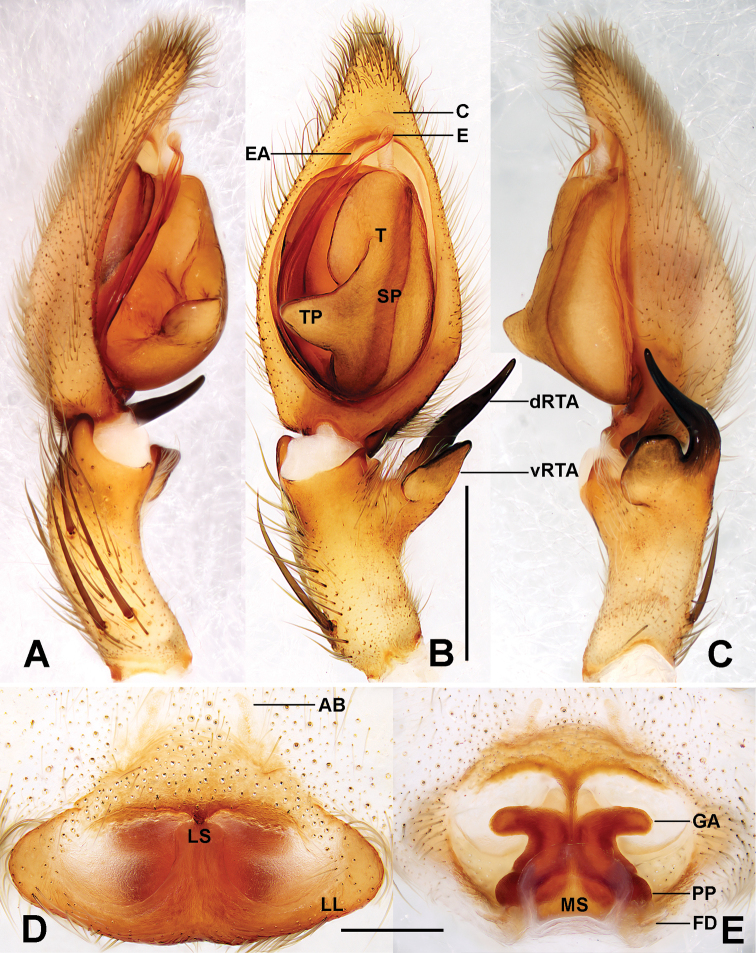
*Sinopodawuyiensis* Liu, 2021 **A–C** palp, left, ventral view **D** epigyne **E** vulva (**A** prolateral view **B, D** ventral view **C** retrolateral view **E** dorsal view). Abbreviations: AB – anterior bands, C – conductor, dRTA – dorsal branch of RTA, E – embolus, EA – embolic apophysis, FD – fertilisation duct, GA – glandular appendage, LL – lateral lobes, LS – lobal septum, MS – membranous sac, PP – posterior part of spermathecae, SP – spermophore, T – tegulum, TP – tegular protrusion, vRTA – ventral branch of RTA. Scale bars: 0.5 mm.

**Figure 4.**
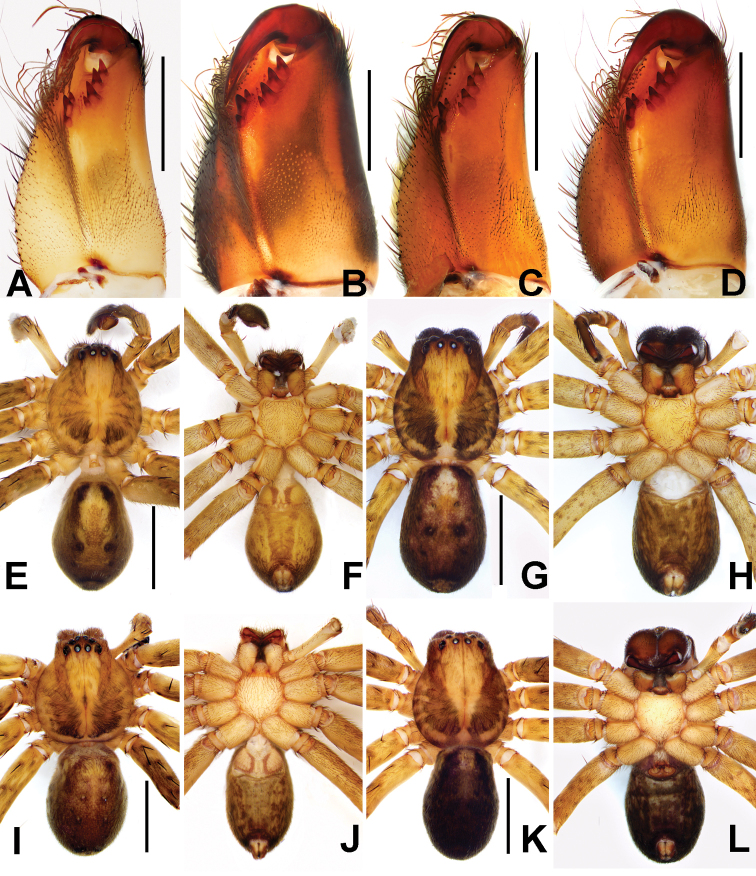
**A, B** cheliceral dentition of *Sinopodamuyuensis* sp. nov. **C, D** cheliceral dentition of *Sinopodawuyiensis* Liu, 2021 **E–H** habitus of *Sinopodamuyuensis* sp. nov. **I–L** habitus of *Sinopodawuyiensis* Liu, 2021 (**A, C, F, J** male, ventral view **E, I** male, dorsal view **B, D, H, L** female, ventral view **G, K** female, dorsal view). Scale bars: 0.5 mm (**A–D**); 5 mm (**E–L**).

**Figure 5.**
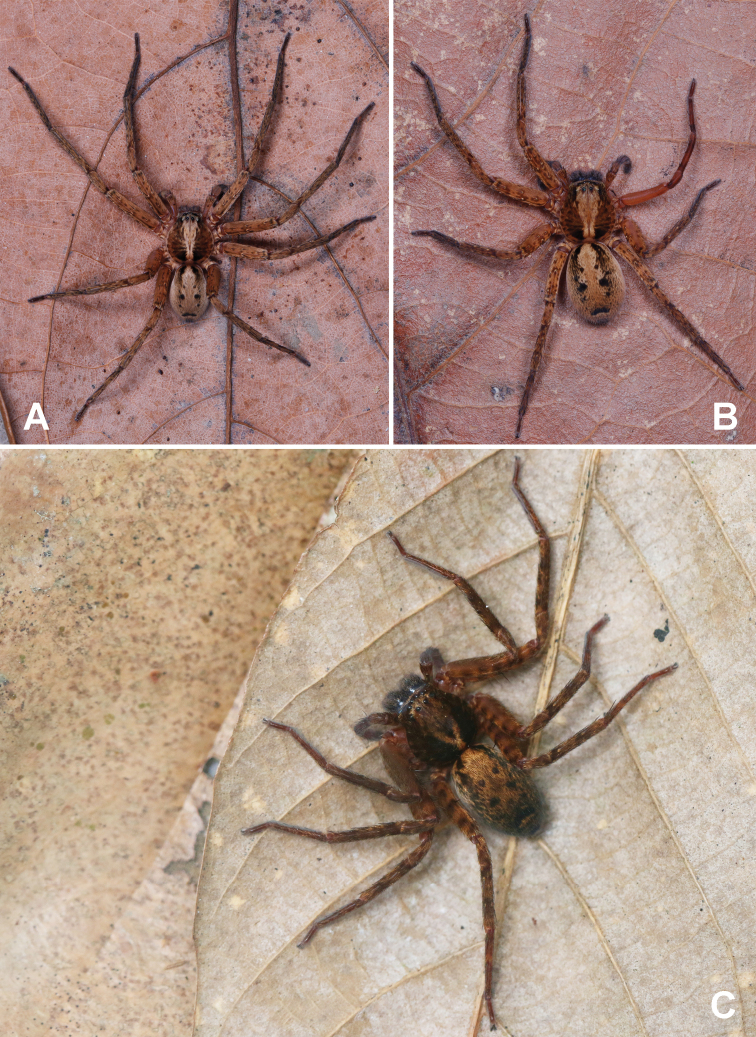
**A, B** Photograph of living *Sinopodamuyuensis* sp. nov. **C** photograph of living *Sinopodawuyiensis* Liu, 2021 **A** male **B, C** female.

**Figure 6.**
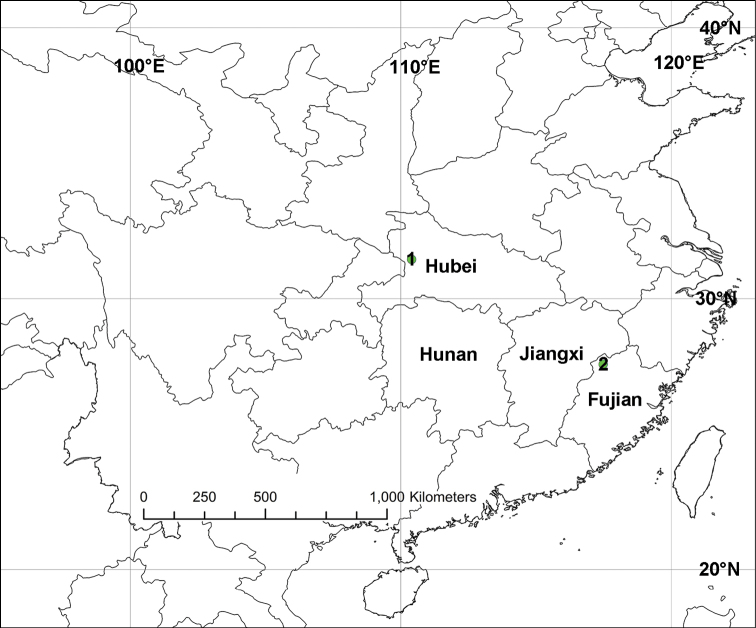
Distribution records of *Sinopoda* species in China **1***Sinopodamuyuensis* sp. nov. **2***Sinopodawuyiensis* Liu, 2021.

Figs 3 , 4C, D, I–L , 5C , 6

Sinopoda wuyiensis Liu, in Zhang et al. 2021: 20, fig. 9A–D (holotype female from Wuyishan National Nature Reserve, Fujian Province, deposited in College of Life Science, Hubei University LJ-202002-ZY, examined)

#### Material examined.

3♂, 4♀ (HUST 0004), **China: *Fujian***: Wuyishan National Reserve, Guadun Village; 27.58°N, 117.48°E; 16.XI.2021; Y. Zhong leg.

#### Diagnosis.

Males of *S.wuyiensis* can be distinguished from other *Sinopoda* species by the combination of the following characters: bases of the tegulum with a distinct sub-triangular protrusion, embolic apophysis reduced, distinctly narrower and shorter than the embolus, and the dRTA about twice as long as the vRTA in ventral view. Females of *S.wuyiensis* may be recognised by the following combination of characters: epigynal field almost fusiform, lateral lobes fused without visible seam, with their anterior and posterior margins almost parallel, and glandular appendages laterad, as wide as posterior part of spermathecae (Fig. 3).

#### Description.

**Male.**PL 4.5, PW 4.3, AW 2.5, OL 5.3, OW 3.0. Eyes: AME 0.18, ALE 0.28, PME 0.26, PLE 0.31, AME–AME 0.24, AME–ALE 0.10, PME–PME 0.24, PME–PLE 0.28, AME–PME 0.33, ALE–PLE 0.23, CHAME 0.10, CHALE 0.14. Setation: Palp: 131, 101, 1021; Fe: I–III 323, IV 321; Pa: I–IV 000; Ti: I–IV 2226; Mt: I 1014, II 2024, III–IV 3036. Measurements of palp and legs: Palp 6.4 (1.9, 1.2, 1.4, –, 1.9), I 18.8 (5.1, 2.2, 5.5, 4.7, 1.3), II 21.9 (6.0, 2.2, 6.2, 5.9, 1.6), III 16.8 (5.1, 2.0, 4.6, 3.6, 1.5), IV 18.3 (4.6, 2.1, 5.1, 4.9, 1.6). Leg formula: II-I-IV-III. Cheliceral furrow with three anterior and four posterior teeth, and with~ 38 denticles (Fig. 4C). Carapace deep yellowish brown dorsally, laterally and posteriorly with dark brown U-shaped pattern, with shallow fovea and radial furrows. Chelicerae deep reddish brown. Sternum yellow with brown margin. Endites and labium deep yellowish brown, with margin deep brown. Legs deep yellowish brown, covered by short spines. Opisthosoma brown dorsally, median part with four brown dots, posteriorly dark brown. Opisthosoma uniformly greyish brown with some brown patches ventrally (Fig. 4I, J).

Palp as in Fig. 3A–C. Cymbium ~ 2× longer than tibia in ventral view. Embolus slightly curved, arising from tegulum at nearly the 6-o’ clock-position in ventral view. Conductor curving distally, arising at 12- to 1-o’clock-position from tegulum. Spermophore almost straight. RTA arising mesially to distally from tibia, with distinct brush of stiff setae. vRTA wider than dRTA, dRTA long, in ventral view proximal part wide and tip tapering.

**Female.**PL 5.4, PW 4.7, AW 3.0, OL 5.3, OW 3.6. Eyes: AME 0.21, ALE 0.33, PME 0.21, PLE 0.39, AME–AME 0.26, AME–ALE 0.07, PME–PME 0.33, PME–PLE 0.46, AME–PME 0.35, ALE–PLE 0.25, CHAME 0.15, CHALE 0.20. Setation: Palp: 131, 101, 2121 1014; Fe: I–III 323, IV 321; Pa: I–IV 000; Ti: I–III 2026, IV 2226; Mt: I–II 1014, III 2024, IV 3036. Measurements of palp and legs: Palp 5.8 (2.0, 0.6, 1.0, –, 2.2), I 15.7 (4.7, 1.7, 4.1, 3.6, 1.6), II 16.2 (4.8, 1.8, 4.5, 3.7, 1.4), III 13.9 (4.4, 1.9, 3.5, 3.0, 1.1), IV 15.1 (4.4, 1.9, 4.1, 3.6, 1.1). Leg formula: II-I-IV-III. Colouration in ethanol as in males, but opisthosoma distinctly darker dorsally and ventrally (Fig. 4K, L; see Zhang et al. (2021) for others described).

#### Distribution.

Known only from the type locality (Fig. 6).

## Supplementary Material

XML Treatment for Sinopoda

XML Treatment for Sinopoda muyuensis

XML Treatment for Sinopoda wuyiensis
